# The potential functions of ferroptosis on urinary stones: mechanisms and therapeutic implications

**DOI:** 10.3389/fphys.2025.1633468

**Published:** 2025-08-20

**Authors:** Yue Ran, Yuhan Ma, Yuexin Luo, Yajun Ruan

**Affiliations:** ^1^ Second Clinical Department, Tongji Medical College, Huazhong University of Science and Technology, Wuhan, China; ^2^ First Clinic School, Union Hospital, Tongji Medical College, Huazhong University of Science and Technology, Wuhan, China; ^3^ Department of Urology, Tongji Hospital, Tongji Medical College, Huazhong University of Science and Technology, Wuhan, China

**Keywords:** ferroptosis, nephrolithiasis, lipid peroxidation, oxidative stress, autophagy

## Abstract

Ferroptosis is a new type of cell death driven by iron-dependent phospholipid peroxidation, which is regulated by a variety of factors including redox homeostasis, iron metabolism, lipid metabolism, cellular metabolism, and mitochondrial function, and plays an important driving role in the development of various tissues and organ damage and diseases. Kidney stones are a common urological disease characterized by high morbidity and high recurrence rate. Currently available preventive or therapeutic treatments for kidney stones are inadequate to cope with the growing clinical demand, suffering from poor efficacy and a higher risk of postoperative complications. Accumulating experimental evidence has established mechanistic links between ferroptosis and nephrolithiasis pathogenesis, highlighting the promising potential of ferroptosis-based therapeutic strategies in kidney stone treatment. This review delves into the latest advances in ferroptosis research associated with kidney stone formation. We review the latest molecular regulatory mechanisms of ferroptosis associated with kidney stone formation from five aspects and elucidate the physiological functions and pathological roles of these pathways. In the conclusion, we critically analyze the therapeutic potential of targeting key molecular mediators within these pathways, providing strategic insights for developing novel therapeutic interventions that may overcome the limitations of conventional approaches in the future.

## 1 Introduction

Kidney stones are crystalline mineral deposits that are primarily formed in the calyces and pelvis. Calcium-based stones, including calcium oxalate (CaOx) and calcium phosphate (CaP) in pure or mixed phases, are the most prevalent form of nephrolithiasis (Khan et al., 2016). Stone formation results from a cascade of physicochemical processes driven by urinary supersaturation, such as nucleation, growth, aggregation, and retention, often initiated in Randall’s plaques, which serve as nucleation sites (Finlayson, 1978). The global prevalence of nephrolithiasis has increased significantly, with epidemiological studies estimating that nearly 9% of the U.S. population will develop kidney stones during their lifetime (Hill et al., 2022). Clinical manifestations range from hematuria and renal colic to severe complications, such as urinary obstruction, infections, and renal impairment, including both acute kidney injury (AKI) and chronic kidney disease (CKD), depending on the stone location and progression (Sasmaz and Kirpat, 2019; Mulay and Anders, 2017; Ripa et al., 2022; Medina-Escobedo et al., 2022). Current therapeutic approaches include extracorporeal shock wave lithotripsy (SWL; 40%–50% global utilization), ureteroscopy (30%–40%), and percutaneous nephrolithotomy (PCNL; 5%–10%) (Khan et al., 2016). However, the recurrence rates remain high, reaching 50% within 5–10 years and 75% within 20 years of treatment (Siener and Hesse, 2021). Despite advances in basic research, the identification of precise therapeutic targets remains challenging, hindering drug development. The limited progress in developing preventive or therapeutic agents in preclinical and clinical trials underscores the need for comprehensive mechanistic insights into cellular injury during stone formation, which may reveal novel treatment strategies for urolithiasis.

Since Dixon’s initial description of ferroptosis in 2012 as an iron-dependent, non-apoptotic cell death modality driven by lipid reactive oxygen species (ROS) accumulation, this process has been extensively studied in diverse pathological conditions using molecular, morphological, genetic, and immunological approaches (Dixon et al., 2012; Tang et al., 2021). Lipid peroxidation, a hallmark of ferroptosis, is regulated by upstream enzymatic and non-enzymatic reactions, as well as downstream scavenging via the xCT-GSH-GPX4 axis. The non-enzymatic Fenton reaction, associated with iron dysregulation, contributes to ROS generation (Conrad and Pratt, 2019). Enzymatic reactions involve two key lipid-remodeling enzymes: acyl-CoA synthetase long-chain family member 4 (ACSL4) and lysophosphatidylcholine acyltransferase 3 (LPCAT3). These enzymes drive the biosynthesis and modification of phosphatidylethanolamine (PE), a critical phospholipid for ferroptosis (Doll et al., 2017; Dixon et al., 2015), facilitating the remodeling of membrane lipids into polyunsaturated fatty acids (PUFAs) and promoting peroxidation (Kagan et al., 2017). The key downstream regulatory axis of ferroptosis is the cystine/glutamate antiporter (xCT)-glutathione (GSH)-GSH peroxidase 4 (GPX4) pathway, which serves as a central negative regulator. GSH acts as a critical antioxidant and essential cofactor for GPX4, enabling the detoxification of phospholipid peroxides (Dixon et al., 2012; Tang et al., 2021; Dixon et al., 2014). This axis exerts a dual control over ferroptosis by directly and indirectly modulating iron and lipid metabolism. The expression and activity of these regulatory molecules are tightly controlled at the post-transcriptional level during different cellular stages.

**FIGURE 1:**
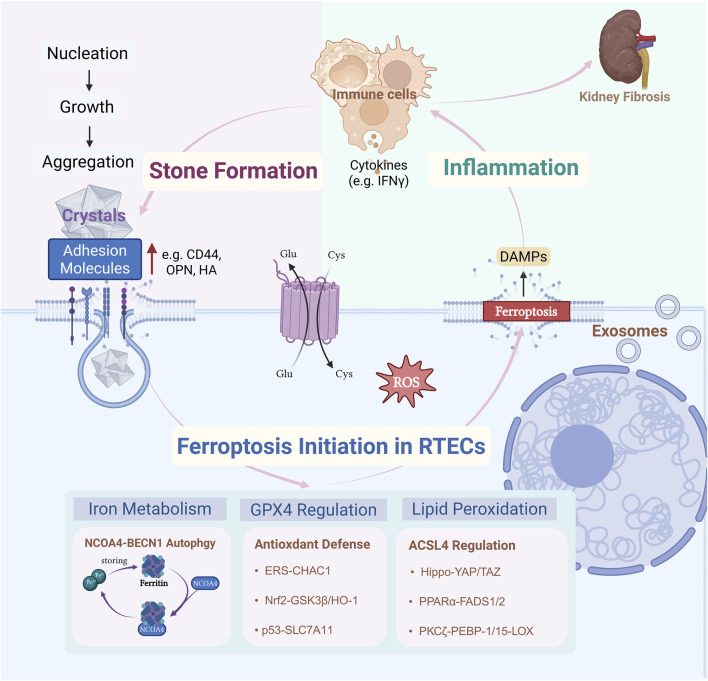
CaOx precipitates when urine is supersaturated, depositing and accumulating on calcified foci of the renal papillae (Randallplaques) or damaged RTECs. This triggers a stress response within RTECs, disrupting iron metabolism through NCOA4-BECN1-mediated autophagy, breaking down GPX4-centered antioxidant defenses, and inducing a lipid-mediated response involving ACSL4peroxidation. These three major mechanisms drive ferroptosis. Ferroptosis in RTECs triggers an immune-inflammatory response in the local microenvironment of the kidney. Inflammatory factors released by various immune cells (e.g., macrophages) act on RTECs, upregulating the expression of adhesion molecules (e.g., CD44, osteoblastogenic protein, and hyaluronic acid) on the surface of iron-death-susceptible RTECs. The recurring cycle of inflammationmay lead to adverse outcomes in renal fibrosis. Created with BioRender.com.

Renal tubular epithelial cell (RTEC) injury is an early pathogenic factor in the formation of urinary stones. High concentrations of CaOx crystals trigger ROS generation and oxidative stress, causing inflammatory damage and altering the fate of RTECs. Furthermore, CaOx crystals activate diverse cellular responses, including autophagy, endoplasmic reticulum stress (ERS), and epithelial-mesenchymal transition (EMT), which synergistically contribute to the pathogenesis of urolithiasis. Recent research has highlighted ferroptosis as a critical process that is interconnected with these pathways. *In vivo* pharmacological experiments by He et al. using the ferroptosis inducer erastin and inhibitor ferrostatin-1 demonstrated that ferroptosis contributes to CaOx stone formation and development via integrated pathophysiological mechanisms (He et al., 2021). Preliminary findings suggest that ferroptosis may be reversible, making it a promising therapeutic target for nephrolithiasis. Despite the growing interest, whether ferroptosis is an initiating factor in stone formation or a secondary consequence of crystal-induced damage remains unknown. We propose that the relationship between ferroptosis and kidney stones is twofold: it can function both as an “active driver” and as a “passive responder.” This review aims to elucidate this critical question from multiple mechanistic perspectives using the most recent experimental evidence. We summarize several core pathways with robust evidence linking ferroptosis and nephrolithiasis, which are discussed in detail below (Figure 1).

## 2 Mechanisms

Ferroptosis is a specific form of stress-induced cell death in RTECs that responds to different stimuli. The core factors driving ferroptosis are GPX4 dysfunction, iron overload, and oxidative stress. Ferroptosis in RTECs can drive kidney stone formation by creating conditions favorable for the development of stone cores. During ferroptosis, uncontrolled lipid peroxidation disrupts the integrity of the cell membrane, leading to the release of intracellular contents. This triggers local inflammatory responses and reshapes the renal microenvironment. These inflammatory and microenvironmental alterations not only exacerbate RTEC injury but also promote crystal aggregation, adhesion, and deposition, thereby accelerating the initiation and progression of nephrolithiasis. This process forms a vicious cycle of “cellular injury–inflammation–crystal deposition.” Ferroptosis also mediates the downstream biological effects of injury induced by various stone crystals. Persistent mechanical damage and inflammation caused by stone deposition elicit local stress responses in RTECs. This results in an imbalance in iron homeostasis and a lower ferroptosis threshold, aggravating renal tissue injury and chronic inflammation. Consequently, a positive feedback loop is established between stone formation and ferroptosis, driving the progression of kidney disease.

During the initiation and progression of kidney stones, the three core mechanisms of ferroptosis participate in related pathological processes in a coordinated and stage-dependent manner. In the early stages of nephrolithiasis, Beclin-1/ATG6 (BECN1)-nuclear receptor coactivator 4 (NCOA4)-mediated ferritinophagy disrupts iron homeostasis, resulting in iron overload. This provides abundant iron substrates for lipid peroxidation and promotes crystal nucleation and deposition. Concurrently, activation of the ACSL4-GPX4 axis drives phospholipid peroxidation and compromises cell membrane integrity, establishing a molecular foundation for early stone development. As stone formation progresses to the late injury stage, dysfunction of the xCT-GSH axis and Nrf2/HO-1 pathway causes the collapse of the antioxidant defense system. This increases the sensitivity of RTECs to ferroptosis, exacerbating cellular injury and inflammation. Ultimately, a vicious cycle is established, driving the persistent progression of stone-related renal damage.

## 3 NCOA4-related iron metabolic disorder

**FIGURE 2:**
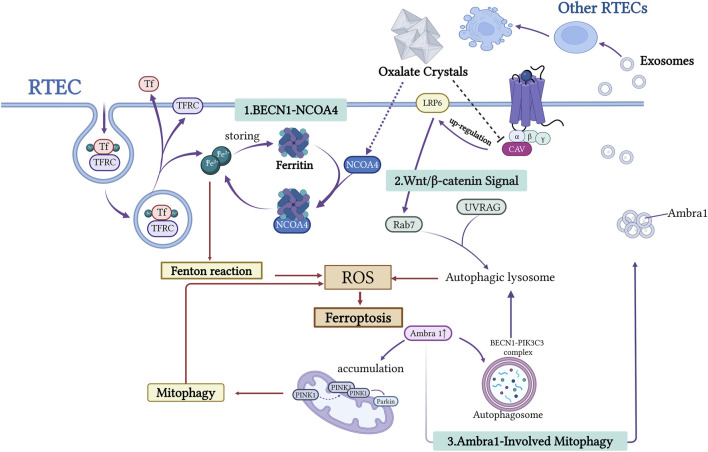
This figure illustrates the mechanism underlying the interaction between autophagy and iron depletion in the formation of urinary stones. RTECs release free Fe^2+^ through the BECN1-NCOA4-mediated ferritin autophagy pathway. This process generates ROS via the Fenton reaction, exacerbating lipid peroxidation and inducing cell ferroptosis. Wnt/β-catenin signaling influences autophagic lysosome formation by regulating Rab7 and UVRAG expression. Ambra1, a key autophagy regulator, promotes autophagosome formation via the BECN1-PIK3C3 complex. Damaged RTECs release exosomes carrying molecules such as Ambra1, which transmits pro-ferroptotic signals to neighboring cells. This network analysis elucidates the central role of autophagy-dependent ferroptosis in stone-associated kidney injury. Created with BioRender.com.

RTECs regulate iron homeostasis through coordinated uptake, storage, and export. Iron is primarily taken up via transferrin receptors (TFR), whereas excess iron is exported by ferroportin (FPN) to maintain a dynamic balance (Davaanyam et al., 2023). Surplus iron is stored as ferritin to prevent oxidative stress caused by free iron (Fuhrmann et al., 2020). When required, iron can be released from ferritin through NCOA4-mediated ferritinophagy, supporting cellular metabolism and mitochondrial function (Hou et al., 2016). Disruption of iron metabolism, such as increased iron uptake or impaired iron export, leads to elevated free intracellular iron levels. This excess free iron triggers ROS production via the Fenton reaction, resulting in lipid peroxidation and ferroptosis, and exacerbating tubular epithelial cell injury (Liang et al., 2022; Maus et al., 2023; Zhao et al., 2023) (Figure 2).

Recent studies have demonstrated that NCOA4-mediated autophagy is the primary pathway for the degradation of ferritin. The autophagy regulator BECN1 works with NCOA4 to promote ferritin breakdown and iron release. BECN1, a central regulator of autophagy initiation, serves as a reliable indicator of autophagy activation when upregulated (Cicchini et al., 2014). As the core component of the BECN1-PIK3C3-PIK3R4 complex, BECN1 orchestrates autophagosome formation and vesicular trafficking (Han et al., 2018; Xia et al., 2014). Evidence suggests that BECN1 plays multiple roles in ferroptosis regulation, particularly by inhibiting xCT, revealing a mechanistic link between autophagy and ferroptosis (Guo et al., 2019; Tan et al., 2022; Su et al., 2023; Lee et al., 2022; Song et al., 2018). NCOA4, a selective cargo receptor, mediates ferritin phagocytosis, thereby modulating iron release, storage, and homeostasis (Bogdan et al., 2016). In response to cellular iron demands, NCOA4 directs ferritin towards lysosomal degradation, facilitating iron liberation. This selective autophagy pathway tightly controls intracellular iron levels by regulating ferritin turnover (Mancias et al., 2014). Song et al. demonstrated that CaOx induces ferroptosis in RTECs by activating BECN1-NCOA4-mediated ferritin autophagy (Song et al., 2021). In animal models of kidney stones, upregulated NCOA4 expression and increased iron pools are closely associated with tubular injury, potentially contributing to the formation of a lithogenic microenvironment (Jin et al., 2023). These findings indicate that ferritin autophagy is a critical mechanistic link between autophagy and ferroptosis in kidney stone formation.

## 4 ACSL4-driven lipid peroxidation

**FIGURE 3:**
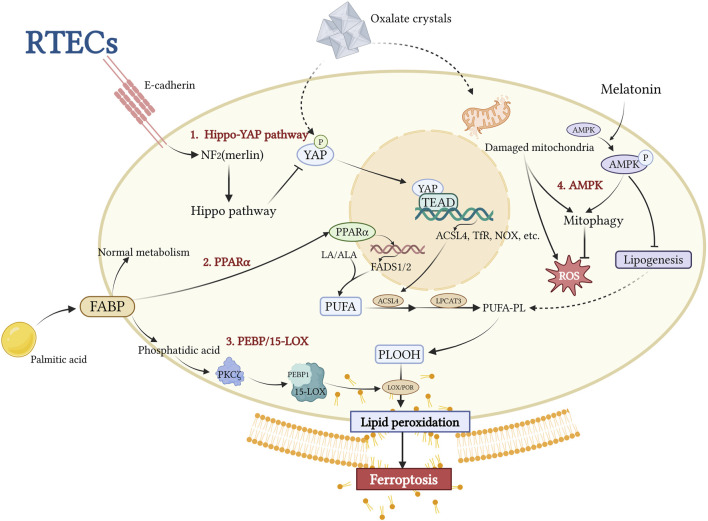
This figure illustrates the central mechanism underlying ACSL4-driven lipid peroxidation in RTECs during stone-induced iron-related cell death. CaOx crystals inhibit the Hippo-YAP pathway, activating the YAP-TEAD complex, which upregulates ACSL4 expression and promotes esterification of PUFAs to membrane phospholipids (PUFA-PL). The PPARα-FADS1/2 axis and the PEBP1/15-LOX pathway collectively promote phospholipid peroxidation (PLOOH) accumulation through lipid metabolic reprogramming and nonclassical lipid peroxidation pathways, respectively. Melatonin inhibits lipid synthesis by activating AMPK-PINK1/Parkin-mediated mitochondrial autophagy, resulting in protective effects. This network analysis revealed ACSL4 as a hub integrating multiple lipid metabolic pathways, ultimately leading to the disruption of membrane integrity and iron-related cell death. Created with BioRender.com.

Lipid peroxidation is the key molecular mechanism of ferroptosis. Untargeted metabolomics studies have revealed that palmitic acid (PA), a major regulator of intracellular free fatty acid (FFA) metabolism, is the only FFA that is significantly upregulated in patients with CaOx kidney stones compared to healthy controls (Wang et al., 2024a). Several experimentally validated pathways contribute to this process, including the Hippo-YAP/TAZ (36), PPARα-FADS1/2, PKC-PEBP-1/15-LOX (35), and AMPK signaling pathways (Figure 3). These canonical pathways collectively drive lipid metabolism toward the enhanced synthesis of PUFA-containing phospholipids (PUFA-PLs).

Among these regulators, ACSL4 has been identified as a pathologically upregulated molecule in both *in vivo* and *in vitro* studies of kidney stone disease. ACSL4-driven lipid peroxidation is a key factor in promoting crystal adhesion, deposition, and ferroptosis-mediated cellular injury in the kidney-stone microenvironment. This is supported by evidence from animal models showing that specific ferroptosis inhibitors reduce the expression of cell adhesion molecules, decrease the levels of ferroptosis-related proteins, and restore cell viability (Wang et al., 2024a). Notably, specific inhibition of ACSL4 by Abemaciclib reduces crystal deposition (Li et al., 2023), whereas the broad ferroptosis inhibitor Ferrostatin-1, which also downregulates ACSL4 expression, primarily alleviates tissue injury (He et al., 2021). These findings strongly support the critical role of ACSL4-mediated lipid peroxidation in the initiation, progression, and injury associated with kidney stone disease. In the following sections, we will focus on the upstream regulatory mechanisms of ACSL4, including the Hippo-YAP/TAZ Pathway and PPARα-FADS1/2 axis, as well as the recently discovered auxiliary regulatory pathways represented by LOX, which synergize with ACSL4 in regulating lipid metabolism.

### 4.1 Hippo-YAP/TAZ pathway

The Hippo pathway, an evolutionarily conserved signaling cascade, plays an essential role in maintaining epithelial homeostasis and regulating immune responses (Dey et al., 2020). Recent studies have implicated it as a critical modulator of ferroptosis (Jiang et al., 2021). Yes-associated protein (YAP), a transcriptional co-activator and central effector of the Hippo signaling pathway (Koo and Guan, 2018), directly regulates ACSL4 expression (Jiang et al., 2021). TAZ, a YAP homolog containing a PDZ-binding motif, functions as a transcriptional co-activator, with both proteins exhibiting cell type-specific expression patterns (Yang et al., 2019). In healthy epithelial cells, E-cadherin-mediated activation of the Hippo-YAP pathway suppresses YAP/TAZ activity, leading to ACSL4 downregulation (Liang et al., 2022). This regulatory mechanism confers ferroptosis resistance to normal RTECs (Liang et al., 2022) (Figure 3). Experimental evidence has demonstrated that YAP promotes ferroptosis through ACSL4 upregulation, exacerbating CaOx deposition and CaOx crystal-induced renal fibrosis (Li et al., 2023). Possible mechanisms underlying renal fibrosis in response to CaOx crystal-induced injury are proposed and discussed in the following sections. Emerging evidence has revealed cell density-dependent regulation of ferroptosis, with high-density cell cultures demonstrating increased resistance to both cysteine deprivation and GPX4 inhibition-induced ferroptosis (Fu et al., 2022; Wu et al., 2019). This density-dependent regulation has been observed in E-cadherin-negative mesenchymal cells (Wu et al., 2019), suggesting a potential connection between crystal deposition-induced EMT in RTECs and the subsequent development of renal fibrosis (Cruz-Solbes and Youker, 2017). As an alternative downstream effector of the Hippo pathway, TAZ has been implicated in multiple pro-fibrotic signaling pathways, including the transforming growth factor-beta (TGF-β) pathway associated with EMT (37).

Although the role of ACSL4 in the continuous formation of kidney stones and tissue injury is well established, experimental evidence for the involvement of the Hippo-YAP/TAZ pathway is limited to ferroptosis-mediated renal injury and stone-induced fibrosis caused by stones. Few studies have supported the direct regulation of stone formation by the Hippo-YAP/TAZ pathway. Moreover, the potential contribution of TAZ to renal stone formation and associated fibrotic processes remains unexplored and warrants further investigation. Although preclinical findings on targeting lipid peroxidation in nephrolithiasis are promising, two major clinical challenges remain. First, a time-specific effect: ACSL4 inhibitors may require early administration (before crystal formation) for optimal efficacy. Second, metabolic uncoupling: Inhibition of lipid peroxidation alone does not address urinary supersaturation (such as in hyperoxaluria). Therefore, combination therapies, such as citrate supplementation, may be necessary to improve the clinical outcomes.

### 4.2 PPARα-FADS1/2 axis

Long-chain PUFAs (LC-PUFAs) are synthesized from linoleic acid (LA) or α-linolenic acid (ALA) via fatty acid desaturases 1 and 2 (FADS1/2). Peroxisome proliferator-activated receptor alpha (PPARα), a nuclear receptor family member and key metabolic sensor, regulates systemic fatty acid metabolism (Montaigne et al., 2021). It is a crucial regulatory receptor in the metabolic microenvironment involved in kidney stone formation. In RTECs, PA, which is central to cellular lipid metabolism, increases cytoplasmic saturated glycerolipids and induces transcriptional stress responses (Koletzko et al., 2019; Athinarayanan et al., 2021). PA exposure upregulates PPARα expression, activating FADS1/2 and promoting the biosynthesis of PUFAs, such as arachidonic acid. The PPARα antagonist GW6471 effectively inhibits PA-induced upregulation of FADS1/2 and PUFA production in animal models (Wang et al., 2024a). ACSL4 esterifies PUFAs into membrane phospholipids, which serve as primary substrates for lipid peroxidation. Consequently, PA has been identified as a “stone architect,” that actively contributes to kidney stone formation (Wang et al., 2024a). Members of the PPAR family have divergent roles in renal pathophysiology. Liu et al. demonstrated that PPARγ activation, unlike PPARα activation, protects against CaOx nephrolithiasis by modulating mitochondrial dynamics in renal tubular cells (Liu et al., 2024). However, the mechanisms underlying these differential effects, particularly their involvement in ferroptosis, remain to be elucidated.

### 4.3 PKCζ-PEBP-1/15-LOX pathway

Recent studies have identified a non-canonical ferroptosis pathway that is distinct from the classical iron-dependent Fenton reaction mechanism. This alternative pathway, activated by ferroptosis suppressor protein 1 (FSP1) inhibition, is mediated by lipoxygenases (LOXs) (Jiang et al., 2021). LOXs catalyze the peroxidation of PUFAs and PUFA-containing membrane phospholipids (Kuhn et al., 2015). Genetic evidence from Alox15 and Alox12 knockdown studies further confirmed the ability of LOXs to induce ferroptosis (Chu et al., 2019; van Leyen et al., 2006; Jin et al., 2008). PE-binding protein 1 (PEBP1), an endogenous RAF1 inhibitor, plays a crucial role in this process. Wang et al. demonstrated that in a CaOx crystal-stimulated microenvironment, protein kinase C ζ (PKCζ) is activated by phosphatidic acid derived from PA overload metabolism. This activation leads to PEBP1 phosphorylation, which relieves its inhibition of RAF1, enables PEBP1 to interact with 15-LOX, and catalyzes the peroxidation of membrane phospholipids. This process acts synergistically with ACSL4-mediated lipid remodeling, exacerbating ferroptosis (Wenzel et al., 2017). Notably, pretreatment with ferrostatin-1 (Fer-1) or the PKCζ-specific inhibitor ζ-Stat significantly reversed these cellular effects, further substantiating this mechanistic pathway (Wang et al., 2024a).

Collectively, both the PPARα–FADS1/2 and PKCζ–PEBP1/15-LOX pathways are activated under conditions of dysregulated cellular lipid metabolism, such as PA overload. The resulting intensified lipid peroxidation promotes CaOx crystal formation and aggravates the renal tissue injury.

## 5 GPX4-centered imbalance of oxidative stress and antioxidant defense

**FIGURE 4:**
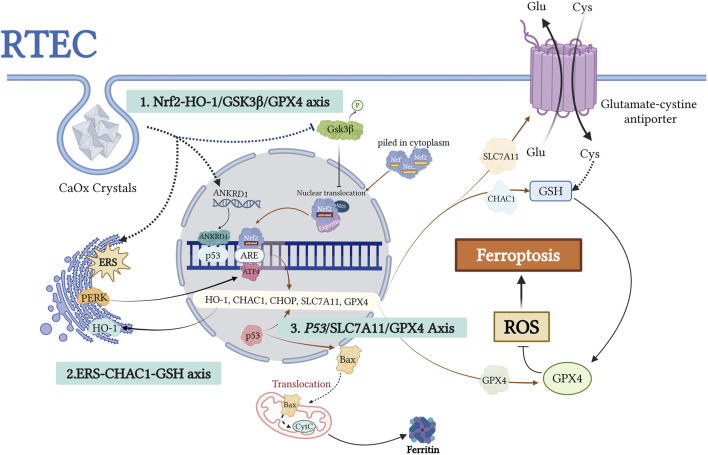
This figure illustrates the molecular mechanism of ferroptosis induced by urolithiasis due to the dysregulation of the GPX4-centered antioxidant defense system. CaOx crystals impairantioxidant gene expression, such as HO-1 and GPX4, through two mechanisms: activation of the PERK/ATF4/CHOP ERS pathwayand inhibition of Nrf2 nuclear translocation (regulated by GSK3β). p53 acetylation inhibits SLC7A11-mediated cystine uptake, leading to GSH depletion and GPX4 inactivation. ANKRD1, a p53 coactivator, synergistically inhibits SLC7A11 and promotes Baxmitochondrial translocation, thereby amplifying oxidative damage. This network demonstrates how GPX4 dysfunction triggers lipidROS accumulation via Nrf2-p53 crosstalk, ultimately causing ferroptosis. The figure highlights the potential targets within the antioxidant pathway for preventing kidney stone formation. Created with BioRender.com.

Ferroptosis, an iron-dependent form of regulated cell death, is characterized by GSH depletion and GPX4 inactivation, both of which are key components of the cellular antioxidant defense system (Hirschhorn and Stockwell, 2019; Mou et al., 2019). In the pathogenesis of CaOx nephrolithiasis, an imbalance between oxidative stress and antioxidant defense drives crystal deposition and renal injury. GPX4 serves as a central node in this process, with its functional collapse resulting from the synergistic action of three major upstream pathways: the Nrf2–GSK3β/GPX4 axis, p53/SLC7A11/GPX4 axis, and ERS–CHAC1/GSH axis. These pathways collectively mediate oxidative membrane damage, promoting crystal nucleation, cell adhesion, and injury to tubular epithelial cells (Figure 4). Among these, Nrf2-GPX4 collapse contributes to the long-term consequences of ferroptosis in RTECs, leading to renal fibrosis. Recent findings have highlighted the reduction in GSH levels induced by ERS, while the role of the P53/SLC7A11/GPX4 axis has been recognized in several studies. As the terminal effector molecule shared by these three regulatory pathways, GPX4 plays a central role in the maintenance of redox homeostasis. In the following sections, we review the three key upstream mechanisms that govern GPX4 regulation.

### 5.1 Nrf2-HO-1/GSK3β/GPX4 axis

Disruption of the Nrf2–GPX4 axis is a key driver of oxidative stress in urolithiasis. Nuclear factor erythroid 2-related factor 2 (Nrf2) regulates the cellular antioxidant defense. Under normal conditions, Nrf2 activity is tightly regulated. However, cellular stress triggers its nuclear translocation and activation of antioxidant response element (ARE)-driven genes, including GPX4 and the xCT cystine/glutamate transporter (Dodson et al., 2019; Osburn et al., 2006; Salazar et al., 2006; Guan et al., 2022; Dong et al., 2023). Nrf2 activation suppresses ferroptosis by upregulating the expression of cytoprotective genes (Hu et al., 2022; Deng et al., 2020). Conversely, GSK3β, a serine/threonine kinase, negatively regulates Nrf2 by promoting cytoplasmic retention and nuclear export (Wei et al., 2022; Liu C. et al., 2022).

In crystal-depositing microenvironments, persistent GSK3β activation under CaOx stress inhibits Nrf2 nuclear translocation, thereby reducing GPX4 and xCT expression. Sustained Nrf2 inhibition results in chronic antioxidant deficiency and accelerates oxidative stress-induced ferroptosis in RTECs, promoting crystal deposition and tissue injury. These effects have been validated in animal and cell models and supported by Nrf2 knockout and Schizandrin B (SchB) intervention studies (Dong et al., 2023).

The Nrf2–HO-1 axis is another crucial regulatory pathway in the cellular antioxidant defense system. Heme oxygenase-1 (HO-1), an enzyme induced by Nrf2 (Saha et al., 2020), plays a key role in scavenging ROS (Ptilovanciv et al., 2013). This pathway protects renal tubular cells against oxidative injury, including ferroptosis (Wang et al., 2022; Li et al., 2021; Jin and Chen, 2022). Zhao et al. demonstrated through *in vivo* and *in vitro* experiments that CaOx crystals can induce ferroptosis via the Nrf2–HO-1 signaling pathway during the formation of CaOx nephrolithiasis (Zhao et al., 2023). This process compromises the resistance of HK-2 cells to oxidative stress and other adverse factors, modulated by ferroptosis regulators such as Fer-1 and erastin. Activation of this pathway exacerbates cellular injury, increases cell–crystal adhesion, and promotes CaOx crystal deposition in the kidney, ultimately contributing to extensive cell–crystal interactions and tissue damage (Zhao et al., 2023).

### 5.2 ERS-CHAC1-GSH axis

The imbalance between oxidative and antioxidative mechanisms plays a crucial role in the interplay between ERS and ferroptosis during the formation of kidney stones. ERS can induce oxidative stress, disrupt calcium homeostasis, and trigger lipid peroxidation, all of which are critical factors in the initiation of ferroptosis (Li et al., 2024; Zhang et al., 2024; Lee et al., 2018). Proteins activated in ERS-induced unfolded protein response (UPR) pathways, such as PERK and ATF6, upregulate CHOP expression, which is a key mediator of ferroptosis. CHAC1, a crucial enzyme for GSH degradation and a downstream molecule in the ATF4-CHOP pathway, is a biomarker for ferroptosis (Xiao et al., 2022; Xu et al., 2023). This upregulation accelerates the depletion of GSH. Additionally, ERS inhibits XCT-mediated cystine uptake and the xCT, thereby reducing intracellular GSH synthesis and diminishing the ability to neutralize lipid peroxides, ultimately increasing sensitivity to ferroptosis (Zhang et al., 2024).

Recent research by Dong et al. demonstrated that under excessive ERS conditions, the PERK/ATF4/CHAC1 pathway of UPR is highly activated in CaOx stone models, exacerbating injury to RTECs via two mechanisms. CHAC1-mediated GSH depletion directly impairs cellular antioxidant capacity, aggravating oxidative stress and inflammatory responses. In contrast, ferroptosis activation promotes plasma membrane rupture and mitochondrial dysfunction, providing more adhesion sites, such as CD44 and ANXA2, for CaOx crystals, thereby accelerating stone formation and establishing a vicious cycle of renal injury and crystal deposition. This mechanism has been validated in both animal models and cellular experiments. Inhibition of ERS or knockdown of CHAC1 significantly restored GSH levels, reduced ferroptosis marker accumulation, and markedly decreased renal fibrosis and crystal deposition, confirming the critical role of the ERS-CHAC1-GSH axis in kidney stone formation (Dong et al., 2025).

### 5.3 *P53*/SLC7A11/GPX4 axis

**FIGURE 5:**
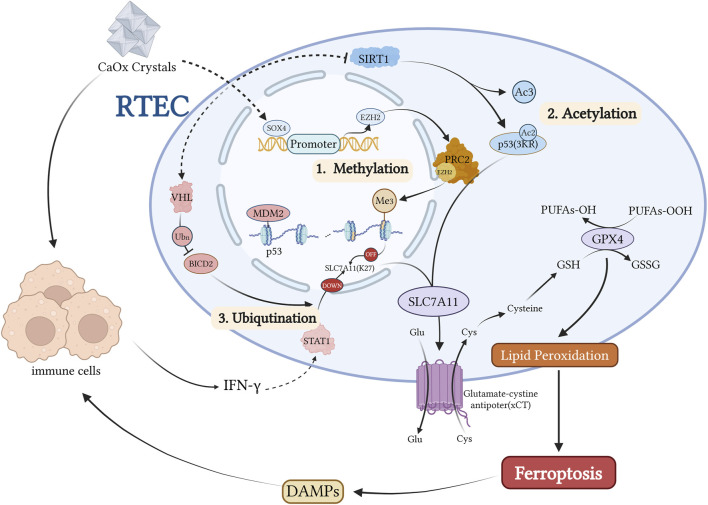
Kidney stones activate the transcription factor SOX4, which directly induces EZH2 expression, a PRC2 methyltransferase. This leads to histone methylation of SLC7A11 and subsequent downregulation of this ferroptosis-protective factor. *P53* is primarily regulated at the post-translational level, including ubiquitination mediated by the MDM2-MDMX complex, the 3 KR acetylation-deficient mutation of *P53*, and modulation of five acetylation sites by the deacetylase SIRT1. During kidney stone formation, SIRT1 activity is inhibited, resulting in increased *P53* acetylation levels and subsequent suppression of *SLC7A11* transcription. Conversely, when SIRT1 activity is normal or *P53* carries a 3 KR acetylation-deficient mutation, the two-site acetylated *P53* fails to suppress *SLC7A11* expression. Additionally, kidney stones induce the activation of the E3 ubiquitin ligase VHL, which downregulates BICD2 levels through ubiquitination, ultimately inhibiting STAT1 nuclear translocation, reducing SLC7A11 expression, and increasing the cellular sensitivity to ferroptosis. Created with BioRender.com.

The tumor suppressor p53 serves as a master regulator of diverse cellular processes, including cell survival, apoptosis and DNA repair (Kruiswijk et al., 2015; Green and Kroemer, 2009; Levine, 2019). Emerging evidence suggests that p53 plays a central role in regulating ferroptosis through its effects on iron metabolism, lipid peroxidation, and the xCT system (Tarangelo et al., 2018; Jiang et al., 2015; Ou et al., 2016; Xie et al., 2017; Zheng and Conrad, 2020). Ye et al. demonstrated reduced Sirt1 expression in the renal tissues of patients with nephrolithiasis. Sirt1, an NAD-dependent deacetylase, modulates ferroptosis by deacetylating p53. Studies involving Sirt1 knockout or overexpression, rescue experiments with p53 3 KR (K117R/K161R/K162R) mutants, ferroptosis inhibitor Lip-1 treatment, and Sirt1-specific agonist SRT1720 administration in animal models consistently support a mechanism in which Sirt1 promotes ferroptosis through p53 deacetylation, thereby mediating CaOx crystal-induced renal fibrosis. Notably, pharmacological activation of Sirt1 in animal models reduces crystal deposition (Ye et al., 2023). CaOx crystal deposition leads to p53 hyperactivation and acetylation, suppressing the transcription of the xCT subunit SLC7A11. This reduces intracellular cystine uptake, limits GSH synthesis, and significantly decreases GPX4 enzymatic activity (He et al., 2021; Xu et al., 2023). Consequently, RTECs become more susceptible to ferroptosis, exposing the basement membrane and crystal adhesion sites (Figure 5). This series of changes creates a cycle in which RTEC ferroptosis and crystal adhesion/deposition reinforce each other, ultimately leading to renal fibrosis. ANKRD1, a p53 co-activator, forms a complex with p53, further inhibiting SLC7A11 transcription, impeding GSH synthesis, and inactivating GPX4. ANKRD1 is upregulated following tissue injury (Samaras et al., 2015), in CaOx-stressed renal tubular cells (Zhao et al., 2023) and is considered a risk factor for ferroptosis in patients with urolithiasis. It facilitates crystal deposition and RTEC ferroptotic injury via the p53/SLC7A11 axis, although further *in vivo* and pathway-dependent experiments are required for confirmation (Zhao et al., 2023). Additionally, ANKRD1 has SLC7A11-independent effects, such as promoting Bax translocation, regulating ferritin deposition following mitochondrial injury, and interacting with the Hippo pathway as a downstream suppressor (Shen et al., 2015; Graupner et al., 2011; Wang et al., 2024b). Recent studies have identified the downregulation of MDM4, a negative regulator of p53, in CaOx-induced RTEC injury. MDM4 suppresses p53 activity and maintains its basal levels (Alarcon-Vargas and Ronai, 2002). Thus, reduced MDM4 expression may enhance p53 activity and increase ferroptosis in renal tubular cells exposed to CaOx crystals (Hou et al., 2024). However, the exact mechanisms underlying its role in stone formation require further investigation to be fully understood.

In summary, CaOx crystals induce p53 hyperactivation and acetylation, suppress SLC7A11 transcription, reduce cystine uptake, impair GSH synthesis, and inactivate GPX4, thereby promoting ferroptosis in RTECs (He et al., 2021). Sirt1 acts as a negative regulator of this process via p53 deacetylation, and its activation mitigates crystal deposition and fibrosis. ANKRD1, a p53 co-activator, synergistically suppresses SLC7A11, establishing a feedback loop between ferroptosis and crystal deposition. This axis represents a critical target for therapeutic intervention in nephrolithiasis.

## 6 Peripheral regulatory pathways in ferroptosis and kidney stone formation

Recent studies have identified several peripheral regulatory pathways involved in ferroptosis during the formation of kidney stones. These include epigenetic modifications, such as histone methylation and post-translational modifications, including acetylation and ubiquitination, which influence ferroptosis sensitivity. Mechanisms such as EZH2-mediated histone methylation and SIRT1-dependent p53 deacetylation have been directly implicated in kidney stone-related renal injury through ferroptosis regulation.

*Epigenetic Modifications*: The SOX4-EZH2-SLC7A11 axis plays a crucial role in CaOx crystal-induced renal injury (Yan et al., 2024) (Figure 5). SOX4 upregulates EZH2, leading to H3K27 trimethylation and epigenetic silencing of SLC7A11, thereby promoting ferroptosis and renal damage (Yan et al., 2024). Both genetic knockdown and pharmacological inhibition of EZH2 have shown protective effects against renal injury and ferroptosis in experimental models (Su et al., 2024).

*Protein Interactions*: CAV1, a membrane scaffolding protein (Nwosu et al., 2016), protects against CaOx-induced renal tubular injury by upregulating LRP6 and activating the Wnt/β-catenin pathway, thereby suppressing autophagy-dependent ferroptosis. CaOx reduces the expression of CAV1, LRP6, and Wnt/β-catenin components, underscoring the protective role of this axis (Yang Y. et al., 2022).

*Exosomal Communication*: Exosomes facilitate intercellular communication by transferring bioactive molecules between renal tubular cells (Noonin and Thongboonkerd, 2021; Liu W. Z. et al., 2022) (Figure 2). AMBRA1, an autophagy-related protein, has been identified as a ferroptosis biomarker in CaOx-induced nephrolithiasis, highlighting the crosstalk between autophagy and ferroptosis in stone-induced kidney injury. Oxalate-treated cells release AMBRA1-enriched exosomes that promote autophagy and ferroptosis in recipient cells via the PINK1/Parkin pathway and by modulating BECN1(91). These effects were reduced when AMBRA1 was knocked down in exosomes, supporting the role of exosomal AMBRA1 in mitophagy and ferroptosis during CaOx-induced injury. However, direct AMBRA1 depletion in HK-2 cells did not significantly affect basal autophagy or ferroptosis, indicating that its function may be cell context-dependent and requires further investigation (Su et al., 2024).

*AMPK Signaling*: Although AMPK is a key energy sensor, its involvement in the regulation of ferroptosis remains controversial. In kidney stone disease models, Zhou et al. found that melatonin enhanced mitophagy and AMPK phosphorylation in both rats and oxalate-treated HK-2 cells (Zhou et al., 2023). Mechanistically, melatonin activates the AMPK-PINK1-Parkin pathway, promoting mitophagy and suppressing oxalate-induced ferroptosis. The protective effects of melatonin are lost when AMPK or PINK1 is inhibited, highlighting this pathway as a potential therapeutic target for kidney stone prevention. These findings emphasize the need for further studies to clarify the stage-specific mechanisms of stone disease.

These findings emphasize the need for further studies to clarify the stage-specific mechanisms of ferroptosis and kidney stone formation.

## 7 Treatment implications

Conventional treatments, such as SWL, ureteroscopic lithotripsy, and PCNL, remain essential for the effective removal of kidney stones in clinical practice. However, these approaches primarily address existing stones and are largely ineffective in preventing the formation of new stones. Recent advances in mechanism-based pharmacological therapies show promise for preventing stone recurrence by targeting the molecular pathways involved in stone pathogenesis (Table 1). These strategies can interrupt the cycles of renal injury, inflammation, and crystal deposition. Specifically, the inhibition of ferroptosis-mediated tubular damage may help prevent disease progression and recurrence. These pharmacological advances have the potential to complement existing surgical interventions and improve the long-term outcomes of patients with kidney stones. However, further studies are required to comprehensively assess the potential nephrotoxicity of these agents before their widespread use in clinical settings.

**TABLE 1:** Potential therapeutic strategies targeting ferroptosis in nephrolithiasis.

Drug/Strategy	Mechanism	Development status	Potential role in nephrolithiasis
Dimethyl fumarate (Zhen et al., 2021)	Nrf2 activator	Approved for Multiple Sclerosis	Reduction in stone-induced damage
Omaveloxolone (Lynch et al., 2021)	Nrf2 activator	Approved for Friedreich’s ataxia	Potential renoprotection
Rosiglitazone (Wang et al., 2016)	PPAR-γ agonist	Approved for Type 2 Diabetes	Renoprotection
Bardoxolone methyl (Wang et al., 2014)	Nrf2 activator	Clinical trials	Reduction of oxidative damage
Pemafibrate (K-877) (Maki et al., 2017)	Selective PPARα modulator	Clinical trials	Renoprotection
Liproxstatin-1 (Shi et al., 2024)	Lipid peroxidation inhibitor	Preclinical (animal)	Renoprotection
XJB-5-131 (Zhao et al., 2020)	Mitochondria-targeted antioxidant	Preclinical (animal)	Reduction of oxidative damage
GW7647 (Qu et al., 2022)	Selective PPARα agonist	Preclinical (*in vitro*)	Potential renoprotection
SC-26196 (Li et al., 2025)	FADS2 inhibitor	Preclinical (*in vitro*)	Potential renoprotection
Luteolin (Ye et al., 2025)	Antioxidant	Preclinical (*in vitro*/animal)	Potential renoprotection
Alda-1 (Zhang et al., 2025)	ALDH2 activator	Preclinical (animal)	Reduction of oxidative damage
CRISPR-dCas13d-eIF4G (He et al., 2024)	Gene editing system	Preclinical (animal)	Reduction in CaOx-induced injury

Dimethyl fumarate (DMF), an oral Nrf2 activator approved for multiple sclerosis treatment, enhances antioxidant defense by activating the Nrf2–GPX4 pathway in the liver. DMF inhibits lipid peroxidation and ferroptosis by upregulating GPX4 expression. Although its clinical use in nephrolithiasis remains unknown, DMF has shown renoprotective effects in preclinical models, suggesting potential to mitigate kidney stone-induced damage (Zhen et al., 2021). Other Nrf2 activators, such as omaveloxolone (OMA), widely used in the treatment of Friedreich ataxia, may exert renoprotective effects through similar mechanisms. However, their efficacy in kidney stone management requires further investigation (Lynch et al., 2021). Rosiglitazone, a PPAR-γ agonist used in type 2 diabetes mellitus management (Balfour and Plosker, 1999), downregulates ACSL4 expression by activating PPAR-γ, thereby reducing PUFA-PE production. In ischemia-reperfusion-induced kidney injury models, rosiglitazone decreases ferroptosis markers and protects renal tissues, providing a potential therapeutic strategy for kidney stone-related renal damage (Wang et al., 2016).

Several therapeutic candidates are currently in clinical trials, indicating progress in the management of kidney stone-related injuries. Pharmacological agents approved for other clinical indications are being investigated for their potential renoprotective effects in nephrolithiasis. Bardoxolone methyl enhances Nrf2 activity by inhibiting Keap1, thereby reducing oxidative stress and kidney injury. It also suppresses the NF-κB pathway, decreasing proinflammatory cytokine production and crystal-induced inflammation (Ruiz et al., 2013; Wang et al., 2014). These effects may help prevent complications of nephrolithiasis. Bardoxolone methyl is currently undergoing clinical trials to evaluate its long-term safety and efficacy in patients with CKD. Pemafibrate (K-877), a novel selective PPARα modulator (SPPARMα) that improves lipid metabolism, has completed phase 3 trials, advancing next-generation fibrate therapy (Maki et al., 2017). A recent case-control study demonstrated that K-877 significantly reduced the risk of cardiovascular events in patients with CKD, highlighting the therapeutic potential of fibrates in kidney disease (Goto et al., 2024).

Several emerging therapeutic agents and strategies remain in the preclinical stage. The ferroptosis inhibitor liproxstatin-1 and mitochondria-targeted antioxidant XJB-5-131 have shown efficacy in animal models of AKI (Yang K. et al., 2022; Chen et al., 2023; Shi et al., 2024; Zhao et al., 2020). GW7647, a selective PPARα agonist, and SC-26196, a FADS1 inhibitor, are currently limited to laboratory studies (Qu et al., 2022; Li et al., 2025). The experimental plant flavonoid luteolin and the ALDH2 activator Alda-1 have demonstrated potential *in vitro* and in animal studies (Ye et al., 2025; Zhang et al., 2025). Gene editing approaches, such as CRISPR-dCas13d-eIF4G, have been investigated in cell and animal models of CaOx-induced kidney injury, but their clinical application is lacking (He et al., 2024). Other novel pathways and targets, including cGAS-STING signaling, DHODH, FSP1, and GSH metabolic regulation, remain in the early stages of experimental investigation (Liang et al., 2023).

## 8 Summary

Ferroptosis, an iron-dependent form of regulated cell death mediated by lipid peroxidation, plays a crucial role in the pathogenesis of kidney stones. Evidence suggests that ferroptosis is associated with renal tubular epithelial injury, oxidative stress, iron and lipid metabolic disorders, and impaired antioxidant defenses. This creates a cycle that promotes crystal adhesion, aggregation, and persistent inflammation in the case of nephrolithiasis. The core mechanisms include BECN1-NCOA4-mediated ferritinophagy, which disrupts iron homeostasis, ACSL4-driven lipid peroxidation, and collapse of the GPX4-centered antioxidant system. Upstream regulation involves the Hippo-YAP/TAZ pathway, PPARα-FADS1/2 axis, PKCζ-PEBP1/15-LOX pathway, and epigenetic modifications such as SOX4-EZH2-SLC7A11 and SIRT1-p53 signaling. Finally, clinical and preclinical evidence highlights the translational potential of emerging therapeutic strategies targeting ferroptosis, including the repurposing of clinically approved drugs and the development of novel preclinical candidates.

## Glossary

CaOx: calcium oxalate
CaP: calcium phosphate
AKI: acute kidney injury
CKD: chronic kidney disease
SWL: shock wave lithotripsy
PCNL: percutaneous nephrolithotomy
ACSL4: Acyl-CoA synthetase long-chain family member 4
LPCAT3: lysophosphatidylcholine acyltransferase 3
PE: phosphatidylethanolamine
PUFAs: polyunsaturated fatty acids
xCT: cystine/glutamate antiporter
GSH: glutathione
GPX4: glutathione peroxidase 4
RTEC: renal tubular epithelial cell
ERS: endoplasmic reticulum stress
EMT: epithelial-mesenchymal transition
TFR: transferrin receptors
ROS: reactive oxygen species
NCOA4: nuclear receptor coactivator 4
PA: palmitic acid
FFA: free fatty acid
PUFA-PLs: polyunsaturated fatty acid-containing phospholipids
YAP: Yes-associated protein
TGF-β: transforming growth factor-β
LC-PUFAs: long-chain polyunsaturated fatty acids
FADS1/2: fatty acid desaturases 1 and 2
PPARα: peroxisome proliferator-activated receptor alpha
FSP1: ferroptosis suppressor protein 1
LOXs: lipoxygenases
PEBP1: phosphatidylethanolamine-binding protein 1
PKCζ: protein kinase C ζ
Nrf2: nuclear factor erythroid 2-related factor 2
ARE: antioxidant response element
HO-1: heme oxygenase-1
UPR: unfolded protein response
DMF: dimethyl fumarate
OMA: omaveloxolone
SPPARMα: selective PPARα.

## References

[B1] Alarcon-Vargas D. Ronai Z. (2002). p53-Mdm2--the affair that never ends. Carcinogenesis 23 (4), 541–547. 10.1093/carcin/23.4.541 11960904

[B2] Athinarayanan S. Fan Y. Y. Wang X. Callaway E. Cai D. Chalasani N. (2021). Fatty acid desaturase 1 influences hepatic lipid homeostasis by modulating the PPARα-FGF21 axis. Hepatol. Commun. 5 (3), 461–477. 10.1002/hep4.1629 33681679 PMC7917273

[B3] Balfour J. A. Plosker G. L. (1999). Rosiglitazone. Drugs 57 (6), 921–930. 10.2165/00003495-199957060-00007 10400405

[B4] Bogdan A. R. Miyazawa M. Hashimoto K. Tsuji Y. (2016). Regulators of iron homeostasis: new players in metabolism, cell death, and disease. Trends Biochem. Sci. 41 (3), 274–286. 10.1016/j.tibs.2015.11.012 26725301 PMC4783254

[B5] Chen F. Kang R. Liu J. Tang D. (2023). The ACSL4 network regulates cell death and autophagy in diseases. Biol. (Basel). 12 (6), 864. 10.3390/biology12060864 PMC10295397 37372148

[B6] Chu B. Kon N. Chen D. Li T. Liu T. Jiang L. (2019). ALOX12 is required for p53-mediated tumour suppression through a distinct ferroptosis pathway. Nat. Cell Biol. 21 (5), 579–591. 10.1038/s41556-019-0305-6 30962574 PMC6624840

[B7] Cicchini M. Chakrabarti R. Kongara S. Price S. Nahar R. Lozy F. (2014). Autophagy regulator BECN1 suppresses mammary tumorigenesis driven by WNT1 activation and following parity. Autophagy 10 (11), 2036–2052. 10.4161/auto.34398 25483966 PMC4502817

[B8] Conrad M. Pratt D. A. (2019). The chemical basis of ferroptosis. Nat. Chem. Biol. 15 (12), 1137–1147. 10.1038/s41589-019-0408-1 31740834

[B9] Cruz-Solbes A. S. Youker K. (2017). Epithelial to mesenchymal transition (EMT) and endothelial to mesenchymal transition (EndMT): Role and implications in kidney fibrosis. Results Probl. Cell Differ. 60, 345–372. 10.1007/978-3-319-51436-9_13 28409352

[B10] Davaanyam D. Lee H. Seol S. I. Oh S. A. Kim S. W. Lee J. K. (2023). HMGB1 induces hepcidin upregulation in astrocytes and causes an acute iron surge and subsequent ferroptosis in the postischemic brain. Exp. Mol. Med. 55 (11), 2402–2416. 10.1038/s12276-023-01111-z 37907744 PMC10689467

[B11] Deng H. F. Yue L. X. Wang N. N. Zhou Y. Q. Zhou W. Liu X. (2020). Mitochondrial iron overload-mediated inhibition of Nrf2-HO-1/GPX4 assisted ALI-induced nephrotoxicity. Front. Pharmacol. 11, 624529. 10.3389/fphar.2020.624529 33584308 PMC7873870

[B12] Dey A. Varelas X. Guan K. L. (2020). Targeting the hippo pathway in cancer, fibrosis, wound healing and regenerative medicine. Nat. Rev. Drug Discov. 19 (7), 480–494. 10.1038/s41573-020-0070-z 32555376 PMC7880238

[B13] Dixon S. J. Lemberg K. M. Lamprecht M. R. Skouta R. Zaitsev E. M. Gleason C. E. (2012). Ferroptosis: an iron-dependent form of nonapoptotic cell death. Cell 149 (5), 1060–1072. 10.1016/j.cell.2012.03.042 22632970 PMC3367386

[B14] Dixon S. J. Patel D. N. Welsch M. Skouta R. Lee E. D. Hayano M. (2014). Pharmacological inhibition of cystine-glutamate exchange induces endoplasmic reticulum stress and ferroptosis. Elife 3, e02523. 10.7554/eLife.02523 24844246 PMC4054777

[B15] Dixon S. J. Winter G. E. Musavi L. S. Lee E. D. Snijder B. Rebsamen M. (2015). Human haploid cell genetics reveals roles for lipid metabolism genes in nonapoptotic cell death. ACS Chem. Biol. 10 (7), 1604–1609. 10.1021/acschembio.5b00245 25965523 PMC4509420

[B16] Dodson M. Castro-Portuguez R. Zhang D. D. (2019). NRF2 plays a critical role in mitigating lipid peroxidation and ferroptosis. Redox Biol. 23, 101107. 10.1016/j.redox.2019.101107 30692038 PMC6859567

[B17] Doll S. Proneth B. Tyurina Y. Y. Panzilius E. Kobayashi S. Ingold I. (2017). ACSL4 dictates ferroptosis sensitivity by shaping cellular lipid composition. Nat. Chem. Biol. 13 (1), 91–98. 10.1038/nchembio.2239 27842070 PMC5610546

[B18] Dong C. Song C. He Z. Song Q. Song T. Liu J. (2023). Protective efficacy of Schizandrin B on ameliorating nephrolithiasis via regulating GSK3β/Nrf2 signaling-mediated ferroptosis *in vivo* and *in vitro* . Int. Immunopharmacol. 117, 110042. 10.1016/j.intimp.2023.110042 36940552

[B19] Dong C. He Z. Liao W. Jiang Q. Song C. Song Q. (2025). CHAC1 mediates endoplasmic reticulum stress-dependent ferroptosis in calcium Oxalate kidney Stone Formation. Adv. Sci. (Weinh) 12 (10), e2403992. 10.1002/advs.202403992 39836526 PMC11905043

[B20] Finlayson B. (1978). Physicochemical aspects of urolithiasis. Kidney Int. 13 (5), 344–360. 10.1038/ki.1978.53 351263

[B21] Fu M. Hu Y. Lan T. Guan K. L. Luo T. Luo M. (2022). The Hippo signalling pathway and its implications in human health and diseases. Signal Transduct. Target Ther. 7 (1), 376. 10.1038/s41392-022-01191-9 36347846 PMC9643504

[B22] Fuhrmann D. C. Mondorf A. Beifuß J. Jung M. Brüne B. (2020). Hypoxia inhibits ferritinophagy, increases mitochondrial ferritin, and protects from ferroptosis. Redox Biol. 36, 101670. 10.1016/j.redox.2020.101670 32810738 PMC7452134

[B23] Goto H. Iseri K. Hida N. (2024). Fibrates and the risk of cardiovascular outcomes in chronic kidney disease patients. Nephrol. Dial. Transpl. 39 (6), 1016–1022. 10.1093/ndt/gfad248 PMC11139516 38012115

[B24] Graupner V. Alexander E. Overkamp T. Rothfuss O. De Laurenzi V. Gillissen B. F. (2011). Differential regulation of the proapoptotic multidomain protein Bak by p53 and p73 at the promoter level. Cell Death Differ. 18 (7), 1130–1139. 10.1038/cdd.2010.179 21233848 PMC3131957

[B25] Green D. R. Kroemer G. (2009). Cytoplasmic functions of the tumour suppressor p53. Nature 458 (7242), 1127–1130. 10.1038/nature07986 19407794 PMC2814168

[B26] Guan S. Zhang R. Zhao Y. Meng Z. Lu J. (2022). 1,3-Dichloro-2-propanol induced ferroptosis through Nrf2/ARE signaling pathway in hepatocytes. Environ. Toxicol. 37 (10), 2515–2528. 10.1002/tox.23615 35870111

[B27] Guo Y. Liu X. Liu D. Li K. Wang C. Liu Y. (2019). Inhibition of BECN1 suppresses lipid peroxidation by increasing System X(c)(-) activity in early brain injury after subarachnoid Hemorrhage. J. Mol. Neurosci. 67 (4), 622–631. 10.1007/s12031-019-01272-5 30719640

[B28] Han T. Guo M. Gan M. Yu B. Tian X. Wang J. B. (2018). TRIM59 regulates autophagy through modulating both the transcription and the ubiquitination of BECN1. Autophagy 14 (12), 2035–2048. 10.1080/15548627.2018.1491493 30231667 PMC6984771

[B29] He Z. Liao W. Song Q. Li B. Liu J. Xiong Y. (2021). Role of ferroptosis induced by a high concentration of calcium oxalate in the formation and development of urolithiasis. Int. J. Mol. Med. 47 (1), 289–301. 10.3892/ijmm.2020.4770 33416117 PMC7723503

[B30] He Z. Song C. Li S. Dong C. Liao W. Xiong Y. (2024). Development and application of the CRISPR-dcas13d-eIF4G translational regulatory System to inhibit ferroptosis in calcium oxalate crystal-induced kidney injury. Adv. Sci. (Weinh) 11 (17), e2309234. 10.1002/advs.202309234 38380498 PMC11077677

[B31] Hill A. J. Basourakos S. P. Lewicki P. Wu X. Arenas-Gallo C. Chuang D. (2022). Incidence of kidney stones in the United States: the continuous National Health and Nutrition Examination Survey. J. Urol. 207 (4), 851–856. 10.1097/JU.0000000000002331 34854755

[B32] Hirschhorn T. Stockwell B. R. (2019). The development of the concept of ferroptosis. Free Radic. Biol. Med. 133, 130–143. 10.1016/j.freeradbiomed.2018.09.043 30268886 PMC6368883

[B33] Hou W. Xie Y. Song X. Sun X. Lotze M. T. Zeh H. J. 3rd (2016). Autophagy promotes ferroptosis by degradation of ferritin. Autophagy 12 (8), 1425–1428. 10.1080/15548627.2016.1187366 27245739 PMC4968231

[B34] Hou C. Zhong B. Gu S. Wang Y. Ji L. (2024). Identification and validation of the biomarkers related to ferroptosis in calcium oxalate nephrolithiasis. Aging (Albany NY) 16 (7), 5987–6007. 10.18632/aging.205684 38536018 PMC11042938

[B35] Hu J. Gu W. Ma N. Fan X. Ci X. (2022). Leonurine alleviates ferroptosis in cisplatin-induced acute kidney injury by activating the Nrf2 signalling pathway. Br. J. Pharmacol. 179 (15), 3991–4009. 10.1111/bph.15834 35303762

[B36] Jiang L. Kon N. Li T. Wang S. J. Su T. Hibshoosh H. (2015). Ferroptosis as a p53-mediated activity during tumour suppression. Nature 520 (7545), 57–62. 10.1038/nature14344 25799988 PMC4455927

[B37] Jiang X. Stockwell B. R. Conrad M. (2021). Ferroptosis: mechanisms, biology and role in disease. Nat. Rev. Mol. Cell Biol. 22 (4), 266–282. 10.1038/s41580-020-00324-8 33495651 PMC8142022

[B38] Jin T. Chen C. (2022). Umbelliferone delays the progression of diabetic nephropathy by inhibiting ferroptosis through activation of the Nrf-2/HO-1 pathway. Food Chem. Toxicol. 163, 112892. 10.1016/j.fct.2022.112892 35278496

[B39] Jin G. Arai K. Murata Y. Wang S. Stins M. F. Lo E. H. (2008). Protecting against cerebrovascular injury: contributions of 12/15-lipoxygenase to edema formation after transient focal ischemia. Stroke 39 (9), 2538–2543. 10.1161/STROKEAHA.108.514927 18635843 PMC2754072

[B40] Jin L. Yu B. Wang H. Shi L. Yang J. Wu L. (2023). STING promotes ferroptosis through NCOA4-dependent ferritinophagy in acute kidney injury. Free Radic. Biol. Med. 208, 348–360. 10.1016/j.freeradbiomed.2023.08.025 37634745

[B41] Kagan V. E. Mao G. Qu F. Angeli J. P. Doll S. Croix C. S. (2017). Oxidized arachidonic and adrenic PEs navigate cells to ferroptosis. Nat. Chem. Biol. 13 (1), 81–90. 10.1038/nchembio.2238 27842066 PMC5506843

[B42] Khan S. R. Pearle M. S. Robertson W. G. Gambaro G. Canales B. K. Doizi S. (2016). Kidney stones. Nat. Rev. Dis. Prim. 2, 16008. 10.1038/nrdp.2016.8 27188687 PMC5685519

[B43] Koletzko B. Reischl E. Tanjung C. Gonzalez-Casanova I. Ramakrishnan U. Meldrum S. (2019). FADS1 and FADS2 polymorphisms modulate Fatty acid metabolism and dietary impact on health. Annu. Rev. Nutr. 39, 21–44. 10.1146/annurev-nutr-082018-124250 31433740

[B44] Koo J. H. Guan K. L. (2018). Interplay between YAP/TAZ and metabolism. Cell Metab. 28 (2), 196–206. 10.1016/j.cmet.2018.07.010 30089241

[B45] Kruiswijk F. Labuschagne C. F. Vousden K. H. (2015). p53 in survival, death and metabolic health: a lifeguard with a licence to kill. Nat. Rev. Mol. Cell Biol. 16 (7), 393–405. 10.1038/nrm4007 26122615

[B46] Kuhn H. Banthiya S. van Leyen K. (2015). Mammalian lipoxygenases and their biological relevance. Biochim. Biophys. Acta 1851 (4), 308–330. 10.1016/j.bbalip.2014.10.002 25316652 PMC4370320

[B47] Lee Y. S. Lee D. H. Choudry H. A. Bartlett D. L. Lee Y. J. (2018). Ferroptosis-Induced endoplasmic reticulum stress: Cross-talk between ferroptosis and apoptosis. Mol. Cancer Res. 16 (7), 1073–1076. 10.1158/1541-7786.MCR-18-0055 29592897 PMC6030493

[B48] Lee J. You J. H. Roh J. L. (2022). Poly(rC)-binding protein 1 represses ferritinophagy-mediated ferroptosis in head and neck cancer. Redox Biol. 51, 102276. 10.1016/j.redox.2022.102276 35290903 PMC8921323

[B49] Levine A. J. (2019). The many faces of p53: something for everyone. J. Mol. Cell Biol. 11 (7), 524–530. 10.1093/jmcb/mjz026 30925588 PMC6736316

[B50] Li S. Zheng L. Zhang J. Liu X. Wu Z. (2021). Inhibition of ferroptosis by up-regulating Nrf2 delayed the progression of diabetic nephropathy. Free Radic. Biol. Med. 162, 435–449. 10.1016/j.freeradbiomed.2020.10.323 33152439

[B51] Li L. Ye Z. Xia Y. Li B. Chen L. Yan X. (2023). YAP/ACSL4 pathway-mediated ferroptosis promotes renal fibrosis in the presence of kidney stones. Biomedicines 11 (10), 2692. 10.3390/biomedicines11102692 37893066 PMC10603838

[B52] Li Y. Li M. Feng S. Xu Q. Zhang X. Xiong X. (2024). Ferroptosis and endoplasmic reticulum stress in ischemic stroke. Neural Regen. Res. 19 (3), 611–618. 10.4103/1673-5374.380870 37721292 PMC10581588

[B53] Li Z. Li W. Zhang C. Wang J. Geng X. Qu B. (2025). Fatty acid desaturase 2 (FADS2) affects the pluripotency of hESCs by regulating energy metabolism. Int. J. Biol. Macromol. 295, 139449. 10.1016/j.ijbiomac.2024.139449 39756764

[B54] Liang D. Minikes A. M. Jiang X. (2022). Ferroptosis at the intersection of lipid metabolism and cellular signaling. Mol. Cell 82 (12), 2215–2227. 10.1016/j.molcel.2022.03.022 35390277 PMC9233073

[B55] Liang J. L. Jin X. K. Zhang S. M. Huang Q. X. Ji P. Deng X. C. (2023). Specific activation of cGAS-STING pathway by nanotherapeutics-mediated ferroptosis evoked endogenous signaling for boosting systemic tumor immunotherapy. Sci. Bull. (Beijing). 68 (6), 622–636. 10.1016/j.scib.2023.02.027 36914548

[B56] Liu C. Li Y. Wang X. (2022). TDAG51-Deficiency podocytes are protected from high-glucose-induced damage through Nrf2 activation via the AKT-GSK-3β pathway. Inflammation 45 (4), 1520–1533. 10.1007/s10753-022-01638-9 35175494

[B57] Liu W. Z. W. Z. Ma Z. J. Kang X. W. (2022). Current status and outlook of advances in exosome isolation. Anal. Bioanal. Chem. 414 (24), 7123–7141. 10.1007/s00216-022-04253-7 35962791 PMC9375199

[B58] Liu J. Liu X. Guo L. Liu X. Gao Q. Wang E. (2024). PPARγ agonist alleviates calcium oxalate nephrolithiasis by regulating mitochondrial dynamics in renal tubular epithelial cell. PLoS One 19 (9), e0310947. 10.1371/journal.pone.0310947 39325731 PMC11426502

[B59] Lynch D. R. Chin M. P. Delatycki M. B. Subramony S. H. Corti M. Hoyle J. C. (2021). Safety and efficacy of omaveloxolone in Friedreich Ataxia (MOXIe Study). Ann. Neurol. 89 (2), 212–225. 10.1002/ana.25934 33068037 PMC7894504

[B60] Maki T. Maeda Y. Sonoda N. Makimura H. Kimura S. Maeno S. (2017). Renoprotective effect of a novel selective PPARα modulator K-877 in db/db mice: a role of diacylglycerol-protein kinase C-NAD(P)H oxidase pathway. Metabolism 71, 33–45. 10.1016/j.metabol.2017.02.013 28521876

[B61] Mancias J. D. Wang X. Gygi S. P. Harper J. W. Kimmelman A. C. (2014). Quantitative proteomics identifies NCOA4 as the cargo receptor mediating ferritinophagy. Nature 509 (7498), 105–109. 10.1038/nature13148 24695223 PMC4180099

[B62] Maus M. López-Polo V. Mateo L. Lafarga M. Aguilera M. De Lama E. (2023). Iron accumulation drives fibrosis, senescence and the senescence-associated secretory phenotype. Nat. Metab. 5 (12), 2111–2130. 10.1038/s42255-023-00928-2 38097808 PMC10730403

[B63] Medina-Escobedo M. Sánchez-Pozos K. Gutiérrez-Solis A. L. Avila-Nava A. González-Rocha L. Lugo R. (2022). Recurrence of nephrolithiasis and surgical events are associated with chronic kidney disease in adult patients. Med. Kaunas. 58 (3), 420. 10.3390/medicina58030420 PMC8954899 35334596

[B64] Montaigne D. Butruille L. Staels B. (2021). PPAR control of metabolism and cardiovascular functions. Nat. Rev. Cardiol. 18 (12), 809–823. 10.1038/s41569-021-00569-6 34127848

[B65] Mou Y. Wang J. Wu J. He D. Zhang C. Duan C. (2019). Ferroptosis, a new form of cell death: opportunities and challenges in cancer. J. Hematol. Oncol. 12 (1), 34. 10.1186/s13045-019-0720-y 30925886 PMC6441206

[B66] Mulay S. R. Anders H. J. (2017). Crystal nephropathies: mechanisms of crystal-induced kidney injury. Nat. Rev. Nephrol. 13 (4), 226–240. 10.1038/nrneph.2017.10 28218266

[B67] Noonin C. Thongboonkerd V. (2021). Exosome-inflammasome crosstalk and their roles in inflammatory responses. Theranostics 11 (9), 4436–4451. 10.7150/thno.54004 33754070 PMC7977448

[B68] Nwosu Z. C. Ebert M. P. Dooley S. Meyer C. (2016). Caveolin-1 in the regulation of cell metabolism: a cancer perspective. Mol. Cancer 15 (1), 71. 10.1186/s12943-016-0558-7 27852311 PMC5112640

[B69] Osburn W. O. Wakabayashi N. Misra V. Nilles T. Biswal S. Trush M. A. (2006). Nrf2 regulates an adaptive response protecting against oxidative damage following diquat-mediated formation of superoxide anion. Arch. Biochem. Biophys. 454 (1), 7–15. 10.1016/j.abb.2006.08.005 16962985 PMC1851923

[B70] Ou Y. Wang S. J. Li D. Chu B. Gu W. (2016). Activation of SAT1 engages polyamine metabolism with p53-mediated ferroptotic responses. Proc. Natl. Acad. Sci. U. S. A. 113 (44), E6806-E6812–e12. 10.1073/pnas.1607152113 27698118 PMC5098629

[B71] Ptilovanciv E. O. Fernandes G. S. Teixeira L. C. Reis L. A. Pessoa E. A. Convento M. B. (2013). Heme oxygenase 1 improves glucoses metabolism and kidney histological alterations in diabetic rats. Diabetol. Metab. Syndr. 5 (1), 3. 10.1186/1758-5996-5-3 23321053 PMC3562196

[B72] Qu X. X. He J. H. Cui Z. Q. Yang T. Sun X. H. (2022). PPAR-α agonist GW7647 protects against oxidative stress and iron deposit via GPx4 in a transgenic mouse model of Alzheimer's diseases. ACS Chem. Neurosci. 13 (2), 207–216. 10.1021/acschemneuro.1c00516 34965724

[B73] Ripa F. Pietropaolo A. Montanari E. Hameed B. M. Z. Gauhar V. Somani B. K. (2022). Association of kidney stones and recurrent UTIs: the chicken and egg situation. A systematic review of literature. Curr. Urol. Rep. 23 (9), 165–174. 10.1007/s11934-022-01103-y 35877059 PMC9492590

[B74] Ruiz S. Pergola P. E. Zager R. A. Vaziri N. D. (2013). Targeting the transcription factor Nrf2 to ameliorate oxidative stress and inflammation in chronic kidney disease. Kidney Int. 83 (6), 1029–1041. 10.1038/ki.2012.439 23325084 PMC3633725

[B75] Saha S. Buttari B. Panieri E. Profumo E. Saso L. (2020). An overview of Nrf2 signaling pathway and its role in inflammation. Molecules 25 (22), 5474. 10.3390/molecules25225474 33238435 PMC7700122

[B76] Salazar M. Rojo A. I. Velasco D. de Sagarra R. M. Cuadrado A. (2006). Glycogen synthase kinase-3beta inhibits the xenobiotic and antioxidant cell response by direct phosphorylation and nuclear exclusion of the transcription factor Nrf2. J. Biol. Chem. 281 (21), 14841–14851. 10.1074/jbc.M513737200 16551619

[B77] Samaras S. E. Almodóvar-García K. Wu N. Yu F. Davidson J. M. (2015). Global deletion of Ankrd1 results in a wound-healing phenotype associated with dermal fibroblast dysfunction. Am. J. Pathol. 185 (1), 96–109. 10.1016/j.ajpath.2014.09.018 25452119 PMC4278243

[B78] Sasmaz M. Kirpat V. (2019). The relationship between the severity of pain and stone size, hydronephrosis and laboratory parameters in renal colic attack. Am. J. Emerg. Med. 37 (11), 2107–2110. 10.1016/j.ajem.2019.06.013 31196585

[B79] Shen L. Chen C. Wei X. Li X. Luo G. Zhang J. (2015). Overexpression of ankyrin repeat domain 1 enhances cardiomyocyte apoptosis by promoting p53 activation and mitochondrial dysfunction in rodents. Clin. Sci. (Lond). 128 (10), 665–678. 10.1042/CS20140586 25511237

[B80] Shi Z. Du Y. Zheng J. Tang W. Liang Q. Zheng Z. (2024). Liproxstatin-1 alleviated Ischemia/reperfusion-induced acute kidney injury via inhibiting ferroptosis. Antioxidants (Basel) 13 (2), 182. 10.3390/antiox13020182 38397780 PMC10886111

[B81] Siener R. Hesse A. (2021). Effect of black tea consumption on urinary risk factors for kidney stone Formation. Nutrients 13 (6), 4434. 10.3390/nu13124434 34959987 PMC8708000

[B82] Song X. Zhu S. Chen P. Hou W. Wen Q. Liu J. (2018). AMPK-Mediated BECN1 phosphorylation promotes ferroptosis by directly blocking System X(c)(-) activity. Curr. Biol. 28 (15), 2388–2399. 10.1016/j.cub.2018.05.094 30057310 PMC6081251

[B83] Song Q. Liao W. Chen X. He Z. Li D. Li B. (2021). Oxalate activates autophagy to induce ferroptosis of renal tubular epithelial cells and participates in the Formation of kidney stones. Oxid. Med. Cell Longev. 2021, 6630343. 10.1155/2021/6630343 34659638 PMC8514920

[B84] Su L. Zhang J. Gomez H. Kellum J. A. Peng Z. (2023). Mitochondria ROS and mitophagy in acute kidney injury. Autophagy 19 (2), 401–414. 10.1080/15548627.2022.2084862 35678504 PMC9851232

[B85] Su X. Song C. He Z. Song Q. Meng L. Dong C. (2024). Ambra1 in exosomes secreted by HK-2 cells damaged by supersaturated oxalate induce mitophagy and autophagy-ferroptosis in normal HK-2 cells to participate in the occurrence of kidney stones. Biochim. Biophys. Acta Mol. Cell Res. 1871 (1), 119604. 10.1016/j.bbamcr.2023.119604 37806389

[B86] Tan Y. Huang Y. Mei R. Mao F. Yang D. Liu J. (2022). HucMSC-derived exosomes delivered BECN1 induces ferroptosis of hepatic stellate cells via regulating the xCT/GPX4 axis. Cell Death Dis. 13 (4), 319. 10.1038/s41419-022-04764-2 35395830 PMC8993870

[B87] Tang D. Chen X. Kang R. Kroemer G. (2021). Ferroptosis: molecular mechanisms and health implications. Cell Res. 31 (2), 107–125. 10.1038/s41422-020-00441-1 33268902 PMC8026611

[B88] Tarangelo A. Magtanong L. Bieging-Rolett K. T. Li Y. Ye J. Attardi L. D. (2018). p53 Suppresses Metabolic Stress-Induced ferroptosis in cancer cells. Cell Rep. 22 (3), 569–575. 10.1016/j.celrep.2017.12.077 29346757 PMC5791910

[B89] van Leyen K. Kim H. Y. Lee S. R. Jin G. Arai K. Lo E. H. (2006). Baicalein and 12/15-lipoxygenase in the ischemic brain. Stroke 37 (12), 3014–3018. 10.1161/01.STR.0000249004.25444.a5 17053180

[B90] Wang Y. Y. Yang Y. X. Zhe H. He Z. X. Zhou S. F. (2014). Bardoxolone methyl (CDDO-Me) as a therapeutic agent: an update on its pharmacokinetic and pharmacodynamic properties. Drug Des. Devel Ther. 8, 2075–2088. 10.2147/DDDT.S68872 PMC4211867 25364233

[B91] Wang S. Dougherty E. J. Danner R. L. (2016). PPARγ signaling and emerging opportunities for improved therapeutics. Pharmacol. Res. 111, 76–85. 10.1016/j.phrs.2016.02.028 27268145 PMC5026568

[B92] Wang S. Zheng Y. Jin S. Fu Y. Liu Y. (2022). Dioscin protects against cisplatin-induced acute kidney injury by reducing ferroptosis and apoptosis through activating Nrf2/HO-1 signaling. Antioxidants (Basel) 11 (12), 2443. 10.3390/antiox11122443 36552651 PMC9774127

[B93] Wang R. Zhang J. Ren H. Qi S. Xie L. Xie H. (2024a). Dysregulated palmitic acid metabolism promotes the formation of renal calcium-oxalate stones through ferroptosis induced by polyunsaturated fatty acids/phosphatidic acid. Cell Mol. Life Sci. 81 (1), 85. 10.1007/s00018-024-05145-y 38345762 PMC10861707

[B94] Wang R. Cheng F. Yang X. (2024b). FTO attenuates the cytotoxicity of cisplatin in KGN granulosa cell-like tumour cells by regulating the Hippo/YAP1 signalling pathway. J. Ovarian Res. 17 (1), 62. 10.1186/s13048-024-01385-5 38491479 PMC10941382

[B95] Wei X. Wu J. Li J. Yang Q. (2022). PLK2 targets GSK3β to protect against cisplatin-induced acute kidney injury. Exp. Cell Res. 417 (1), 113181. 10.1016/j.yexcr.2022.113181 35523306

[B96] Wenzel S. E. Tyurina Y. Y. Zhao J. St Croix C. M. Dar H. H. Mao G. (2017). PEBP1 wardens ferroptosis by enabling lipoxygenase generation of lipid death signals. Cell 171 (3), 628–641. 10.1016/j.cell.2017.09.044 29053969 PMC5683852

[B97] Wu J. Minikes A. M. Gao M. Bian H. Li Y. Stockwell B. R. (2019). Intercellular interaction dictates cancer cell ferroptosis via NF2-YAP signalling. Nature 572 (7769), 402–406. 10.1038/s41586-019-1426-6 31341276 PMC6697195

[B98] Xia P. Wang S. Huang G. Du Y. Zhu P. Li M. (2014). RNF2 is recruited by WASH to ubiquitinate AMBRA1 leading to downregulation of autophagy. Cell Res. 24 (8), 943–958. 10.1038/cr.2014.85 24980959 PMC4123297

[B99] Xiao R. Wang S. Guo J. Liu S. Ding A. Wang G. (2022). Ferroptosis-related gene NOX4, CHAC1 and HIF1A are valid biomarkers for stomach adenocarcinoma. J. Cell Mol. Med. 26 (4), 1183–1193. 10.1111/jcmm.17171 35023280 PMC8831942

[B100] Xie Y. Zhu S. Song X. Sun X. Fan Y. Liu J. (2017). The tumor suppressor p53 limits ferroptosis by blocking DPP4 activity. Cell Rep. 20 (7), 1692–1704. 10.1016/j.celrep.2017.07.055 28813679

[B101] Xu J. Zhao L. Zhang X. Ying K. Zhou R. Cai W. (2023). Salidroside ameliorates acetaminophen-induced acute liver injury through the inhibition of endoplasmic reticulum stress-mediated ferroptosis by activating the AMPK/SIRT1 pathway. Ecotoxicol. Environ. Saf. 262, 115331. 10.1016/j.ecoenv.2023.115331 37556956

[B102] Yan X. Xia Y. Li B. Ye Z. Li L. Yuan T. (2024). The SOX4/EZH2/SLC7A11 signaling axis mediates ferroptosis in calcium oxalate crystal deposition-induced kidney injury. J. Transl. Med. 22 (1), 9. 10.1186/s12967-023-04793-1 38169402 PMC10763321

[B103] Yang W. H. Ding C. C. Sun T. Rupprecht G. Lin C. C. Hsu D. (2019). The hippo pathway effector TAZ regulates ferroptosis in renal cell carcinoma. Cell Rep. 28 (10), 2501–2508. 10.1016/j.celrep.2019.07.107 31484063 PMC10440760

[B104] Yang Y. Y. Hong S. Lu Y. Wang Q. Wang S. Xun Y. (2022). CAV1 alleviated CaOx stones formation via suppressing autophagy-dependent ferroptosis. PeerJ 10, e14033. 10.7717/peerj.14033 36128191 PMC9482765

[B105] Yang K. K. Zeng L. Yuan X. Wang S. Ge A. Xu H. (2022). The mechanism of ferroptosis regulating oxidative stress in ischemic stroke and the regulation mechanism of natural pharmacological active components. Biomed. Pharmacother. 154, 113611. 10.1016/j.biopha.2022.113611 36081288

[B106] Ye Z. Xia Y. Li L. Li B. Chen L. Yu W. (2023). p53 deacetylation alleviates calcium oxalate deposition-induced renal fibrosis by inhibiting ferroptosis. Biomed. Pharmacother. 164, 114925. 10.1016/j.biopha.2023.114925 37236026

[B107] Ye Z. Yang S. Chen L. Yu W. Xia Y. Li B. (2025). Luteolin alleviated calcium oxalate crystal induced kidney injury by inhibiting Nr4a1-mediated ferroptosis. Phytomedicine 136, 156302. 10.1016/j.phymed.2024.156302 39662099

[B108] Zhang P. Zhou C. Ren X. Jing Q. Gao Y. Yang C. (2024). Inhibiting the compensatory elevation of xCT collaborates with disulfiram/copper-induced GSH consumption for Cascade ferroptosis and cuproptosis. Redox Biol. 69, 103007. 10.1016/j.redox.2023.103007 38150993 PMC10788306

[B109] Zhang J. Wang R. Xie L. Ren H. Luo D. Yang Y. (2025). Pharmacological activation of aldehyde dehydrogenase 2 inhibits ferroptosis via SLC7A11/GPX4 axis to reduce kidney stone formation. Eur. J. Pharmacol. 986, 177132. 10.1016/j.ejphar.2024.177132 39547408

[B110] Zhao Z. Wu J. Xu H. Zhou C. Han B. Zhu H. (2020). XJB-5-131 inhibited ferroptosis in tubular epithelial cells after ischemia-reperfusion injury. Cell Death Dis. 11 (8), 629. 10.1038/s41419-020-02871-6 32796819 PMC7429848

[B111] Zhao J. Wu Y. Zhou K. Huang M. Sun Y. Kang J. (2023). Ferroptosis in calcium oxalate kidney stone formation and the possible regulatory mechanism of ANKRD1. Biochim. Biophys. Acta Mol. Cell Res. 1870 (5), 119452. 10.1016/j.bbamcr.2023.119452 36907445

[B112] Zhen X. Jindong L. Yang Z. Yashi R. Wei G. Wei J. (2021). Activation of Nrf2 pathway by dimethyl fumarate attenuates renal ischemia-reperfusion injury. Transpl. Proc. 53 (7), 2133–2139. 10.1016/j.transproceed.2021.07.017 34426023

[B113] Zheng J. Conrad M. (2020). The metabolic underpinnings of ferroptosis. Cell Metab. 32 (6), 920–937. 10.1016/j.cmet.2020.10.011 33217331

[B114] Zhou J. Meng L. He Z. Song Q. Liu J. Su X. (2023). Melatonin exerts a protective effect in ameliorating nephrolithiasis via targeting AMPK/PINK1-Parkin mediated mitophagy and inhibiting ferroptosis *in vivo* and *in vitro* . Int. Immunopharmacol. 124 (Pt A), 110801. 10.1016/j.intimp.2023.110801 37651854

